# Convolution‐based modified Clarkson integration (CMCI) for electron cutout factor calculation

**DOI:** 10.1002/acm2.12267

**Published:** 2018-02-03

**Authors:** Jina Chang, Mu‐Han Lin, Weiguo Lu, Mingli Chen, Steve Jiang

**Affiliations:** ^1^ Department of Radiation Oncology University of Texas Southwestern Medical Center Dallas TX USA

**Keywords:** cutout factor, electron therapy, modified Clarkson integration

## Abstract

Electron therapy is widely used to treat shallow tumors because of its characteristic sharp dose fall‐off beyond a certain range. A customized cutout is typically applied to block radiation to normal tissues. Determining the final monitor unit (MU) for electron treatment requires an output factor for the cutout, which is usually generated by measurement, especially for highly irregular cutouts. However, manual measurement requires a lengthy quality assurance process with possible errors. This work presents an accurate and efficient cutout output factor prediction model, convolution‐based modified Clarkson integration (CMCI), to replace patient‐specific output factor measurement. Like the Clarkson method, we decompose the field into basic sectors. Unlike the Clarkson integration method, we use annular sectors for output factor estimation. This decomposition method allows calculation via convolution. A 2D distribution of fluence is generated, and the output factor at any given point can be obtained. We applied our method to 10 irregularly shaped cutouts for breast patients for 6E, 9E, and 15E beams and compared the results with measurements and the electron Monte Carlo (eMC) calculation using the Eclipse planning system. While both the CMCI and eMC methods showed good agreement with chamber measurements and film measurements in relative distributions at the nominal source to surface distance (SSD) of 100 cm, eMC generated larger errors than the CMCI method at extended SSDs, with up to −9.28% deviations from the measurement for 6E beam. At extended SSD, the mean absolute errors of our method relative to measurements were 0.92 and 1.14, while the errors of eMC were 1.42 and 1.79 for SSD 105 cm and 110 cm, respectively. These results indicate that our method is more accurate than eMC, especially for low‐energy beams, and can be used for MU calculation and as a QA tool for electron therapy.

## 1 INTRODUCTION

Electron beams have been widely used in radiotherapy to escalate or boost dose for superficial lesions, where the fast dose fall‐off feature of electron beams is desirable for reducing radiation damage to distal tissues. However, variations in electron scattering with respect to beam energy, collimation, and treatment distances make it difficult to predict or create a standard dosimetry model for calculating dose per monitor unit (MU) or, equivalently, output factor.

In most clinics, the output factors of irregularly shaped cutouts have to be measured individually at nominal or extended source to surface distance (SSD), while the output factors of simply shaped cutouts are referred to the institutional linear accelerator data book. However, measuring every cutout factor is not practical for busy clinics, and not every clinic can use an electron treatment planning system (TPS) because of capital and workload costs. Moreover, a simple calculation that includes percentage depth dose, SSD, and output factor is needed for secondary independent MU verification. Therefore, a fast and accurate in‐house tool that predicts the electron output factor for both regular and irregularly shaped cutouts is desirable. This work presents an accurate and efficient cutout output factor prediction model to replace time‐consuming patient‐specific output factor measurement.

The dose calculation algorithms adapted by commercial electron treatment planning systems are the pencil beam method^1^, ^2^ and the Monte Carlo simulation method.^3^, ^4^, ^5^ The CadPlan TPS (Varian Medical Systems Inc., Palo Alto, CA, USA) uses a generalized Gaussian pencil beam model to calculate electron dose. It could predict relative cutout output factors for extended SSDs within a clinically acceptable uncertainty (1–2%) for 18E beams. However, lower energy beams, such as 6E and 12E, produce large errors (>10%) in calculated relative output factors for small or extremely elongated rectangular fields.^6^ The Xio eMC TPS (Elekta CMS Software GmbH, Freiburg, Germany) uses a voxel‐based Monte Carlo planning system developed by Kawrakow *et al*.^7^ Edimo *et al*. validated XiO eMC for electron output factors but concluded that it should be used with caution at lower electron energies, as the calculation showed a maximum error of 4.22% compared to measurement.^8^ The electron Monte Carlo (eMC) in Eclipse TPS (Varian Medical Systems, Palo Alto, CA, USA) is a Macro Monte Carlo (MMC)‐based technique developed by Neuenschwander *et al*.^3^ The MMC uses spheres for local simulation and determines electron characteristics after each sphere from the precalculated database. Hu *et al*. reported that the agreement between the Eclipse electron Monte Carlo (eMC) calculations and measurements was within 3% for cutouts greater than 5 × 5 cm and 5% for cutouts smaller than 5 × 5 cm. However, the agreement was significantly poorer for cutouts of 3 × 3 cm, reaching up to 8% error. Therefore, commercial electron treatment planning systems have limited accuracy for calculating cutout output factors at low‐energy and small‐field dimensions (3 cm or less).^9^

In the literature, the methods for predicting cutout output factors for electron beams include the equivalent square,^10^, ^11^ square root,^12^ one‐dimensional,^13^ and sector‐integration methods.^14^ These algorithms predict the output factor of an irregular electron field using a database of parameters based on measurements.

Following the modified Clarkson Integration (MCI) presented by Kung *et al.,* 15 we propose a new model that predicts the cutout output factor via convolution: the convolution‐based modified Clarkson integration (CMCI). The MCI method uses annular fields to estimate scatter components instead of the pie sectors used in the original Clarkson integration method. Our CMCI method further assumes the cutout factors’ invariance under shifts in the cutout shape. Two‐dimensional convolution can integrate all output contributions in the opening area of an electron cutout and make the CMCI method very efficient. We verified CMCI's accuracy by comparing it with the results from eMC, film dosimetry, and ion chamber measurements at nominal and extended SSD using 10 highly irregularly shaped cutouts.

## 2 METHODS

### 2.1 Modified Clarkson integration (MCI) method for intensity‐modulated radiation therapy

Instead of using the pie sectors used in the original Clarkson integration method, the MCI technique models scatter fluence as circularly symmetric about the central axis. It assumes that all beamlets with distance r from the central axis contribute an equal amount of scatter per MU to the point of calculation. The MCI method is summarized in detail in Algorithm 1. In the first step, the fluence grid $f\left(x,y\right)$ from the MLC sequence files is replaced by $f\left(r\right)$ using an azimuthal average. Then, dose from an annular field with inner and outer radii of (r,r+Δr) is determined by subtracting a circular field of radius r from a field of radius r+Δr. Finally, the final dose is calculated by summing the primary dose and the scatter dose. The fluence $f\left(0\right)$ obtained by averaging $f\left(x,y\right)$ over the circular field r≤1 cm for the primary dose.

**Table:** 

Algorithm 1 MCI technique.
At each radius, $f\left(x,y\right)$ is replaced by $f\left(r\right)$ $$f\left(x,y\right)\to f\left(r\right)\equiv \frac{1}{2\pi }\int \int_{x^{2}+y^{2}=r^{2}}f\left(x,y\right)dxdy$$ Calculate dose of an annular field with inner and outer radii of (r,r+Δr). $$\begin{array}{cc} & D(d,\text{annulus})=D(d,r+\Delta r)-D(d,r)\\ & =D_{\mathrm{ref}}\cdot \left(\mathrm{ISF}\right)\cdot f\left(r\right)\cdot \left(S_{p}\left(r+\Delta r\right)\cdot TPR\left(r+\Delta r\right)-S_{p}\left(r\right)\cdot TPR\left(r\right)\right) \end{array}$$ where ISF = inverse square factor, TPR = tissue phantom ratio, and $S_{p}$ = phantom scatter factor. Final dose is a sum of the priory dose and the scattered dose. $$D\left(d\right)=D\left(d,primary\right)+\sum_{\mathrm{annulus}}D\left(d,\text{annulus}\right)$$

### 2.2 Convolution‐based modified Clarkson integration (CMCI) method for electron output factor

Assuming radially isotropic contributions, the CMCI method also uses annular fields to calculate an electron output contribution. CMCI further assumes that the cutout output factor is shift invariant for a given cutout shape. This assumption neglects scattering's angular dependence on off‐axis beams and central beams. However, such negligence is minor, as the opening area of the cutout is most likely around the center beam. These assumptions allow simple modeling of 2D fluence maps for irregular cutouts and efficient calculation via convolution.

The detailed CMCI method is summarized in Algorithm 2. In the first step, the annular output factor (AOF) for an annular field in the radial range (r,r+Δr) is obtained by subtracting a circular field of radius r from a field of radius r+Δr. The output factor of a field is defined as the ratio of dose per MU at the point of interest to dose per MU of the reference cone. The output factors are measured for multiple circular cutouts with radii ranging from 1 cm to the maximal one that fits in the cone. The output factor resulting from each circle is then fitted with a smoothing curve. To generate the 2D convolution output kernel,$OK[n_{1},n_{2}]$, the weight value of the kernel is determined by dividing the AOF at each annular region by the number of pixels (NP). The 2D kernel is a circular symmetry with a resolution of 1 mm per pixel, and each point in the kernel represents the electron output contribution at each pixel position. Finally, the cutout output factor (COF) is calculated by convolution between the cutout shape f[x,y] and the output kernel $OK[n_{1},,n_{2}]$. The size of $OK\left[n_{1},n_{2}\right]$ is M×M pixels, and the indexed $n_{1}$ and $n_{2}$ are used to loop through the rows and columns of $OK\left[n_{1},n_{2}\right]$ to calculate the sum of products.

**Table:** 

Algorithm 2 CMCI technique.
Computing the kernel Output factors, $D\left(r+\Delta r\right)/D_{0}$ and $D\left(r\right)/D_{0}$, from circular fields of radius *r* + Δ*r* and *r,* respectively, are used to determine the AOF, where the dose *D*_0_ at the center of the cone field is used to normalize the dose $D\left(r+\Delta r\right)$ and $D\left(r\right).$ 2D output kernel, $OK\left[n_{1},n_{2}\right]$, can be calculated using AOF: $$\begin{array}{cc} & OK\left[n_{1},n_{2}\right]=\sum_{\mathrm{annulus}}\mathrm{AOF}\left(\mathrm{annulus}\right)/\text{NP}\left(\mathrm{annulus}\right)\\ & \text{where}\\ & \text{AOF}=\text{annular output factor, and NP = number of pixels} \end{array}.$$ Final cutout output factor is calculated by convolution between the cutout shape f[x,y] and the output kernel $OK[n_{1},n_{2}]$ $$\text{COF}\left[x,y\right]=\sum_{n_{1}=-0}^{M-1}\sum_{n_{2}=-0}^{M-1}OK\left[n_{1},n_{2}\right]\cdot f\left[x-n_{1},y-n_{2}\right]$$

We conducted measurements in a Varian EX linear accelerator (Varian Medical Systems, Palo Alto, CA, USA) at 6E, 9E, and 15E energies. The circular electron cutouts were measured using a 0.015 cm^3^ pinpoint ion chamber (PTW, Freiburg, Germany) in a water phantom (1D Scanner™, Sun Nuclear, Melbourne, FL, USA) with a 15 × 15 cm electron applicator cone, then normalized by the measured value of 10 × 10 cm open applicator to obtain the relative output factor. All measurements were carried out with 100 MU, 100 cm SSD, at the respective $d_{\max}$ of individual cutout and energy combinations. We used these measurement values to generate the convolution kernels. The radii of circular cutouts ranged from 1 to 6 cm with 1 cm intervals. We then interpolated and extrapolated the measured output factors at 1 mm intervals to generate the kernel. The same procedures were performed to generate the convolution kernels at the extended SSDs of 105 cm and 110 cm. For test cutouts, we measured using an ion chamber in the water at the center of the largest opening area of the customized cutout that corresponds to the maximum dose, then normalized by the measured value of 10 × 10 cm open applicator at the center to obtain the relative output factor. For highly irregular fields, finding an ion chamber measurement point is challenging. Such measurements are prone to point variability, particularly for narrow and irregular cutout fields where the steep dose gradient exists. To minimize the measurement uncertainty, we measured three times at the center of the largest opening area of the customized cutout with 2D convolution map guidance. This study used this point for chamber measurement as well as calculation with CMCI and the Eclipse TPS to evaluate the algorithm.

### 2.3 Validation and data analysis

We validated the CMCI method by comparing it with an ion chamber measurement and Eclipse eMC calculation. The eMC calculation consisted of an electron transport/dose deposition model and an electron beam phase space model (Initial Phase Space model). The CT images of the Solid Water phantom (Gammex rmi, Middleton, WI, USA) and the DICOM RT format of the digitized electron cutout were imported into Eclipse. The calculation grid size was 2.5 mm, and statistical uncertainty was 1% with 3D Gaussian smoothing. The MU for each plan was fixed at 100 MU, the same as the measurement setting of an ion chamber. The cutout output factor was defined as dose at $d_{\max}$ with a 15 × 15 cm applicator divided by dose with the reference 10 × 10 cm open applicator.

For relative output distribution, the EDR2 film was placed at $d_{\max}$ between Solid Water slabs, and 2D dose distribution at a perpendicular plane to the beam axis at $d_{\max}$ was also exported from the eMC calculation. The evaluation criterion for relative output comparison was the Gamma index passing, defined as $\Gamma \left({\vec{r}}_{e},{\vec{r}}_{r}\right)=\sqrt{\frac{r^{2}\left({\vec{r}}_{e},{\vec{r}}_{r}\right)}{\Delta d^{2}}+\frac{\delta^{2}\left({\vec{r}}_{e},{\vec{r}}_{r}\right)}{\Delta D^{2}}}$ where $r\left({\vec{r}}_{e},{\vec{r}}_{r}\right)$ and $\delta \left({\vec{r}}_{e},{\vec{r}}_{r}\right)$ are a spatial distance and dose difference between evaluated and reference points, and Δd and ΔD are distance‐to‐agreement and dose criteria. The Gamma acceptance criteria were 3 mm for distance‐to‐agreement and 3% for dose difference. The point of maximum dose was used as a normalization point for gamma analysis. Since the dose denominator ∆D for gamma calculations is the percentage value of the maximum measurement point, this normalization point guarantees the best possible global gamma passing rate. Then, we evaluated a correlation between the gamma passing rate and cutout shape complexity using a shape complexity measure: perimeter^2^/area ratio (P2A).

**Figure 1:**
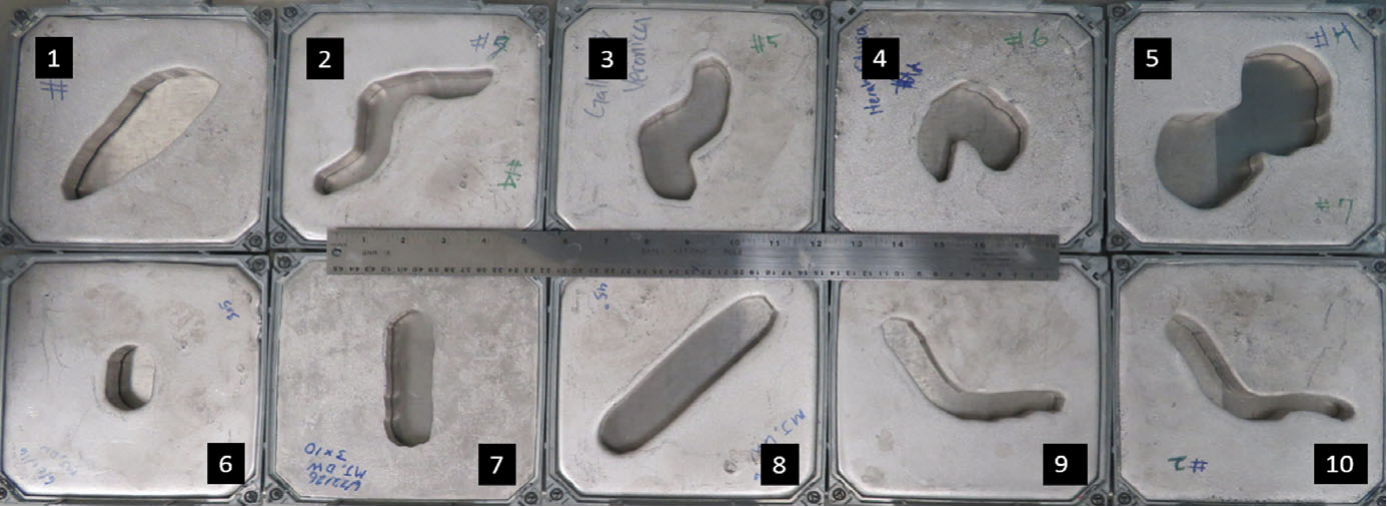
The 10 irregular test cases for 15 × 15 applicator cone.

We tested our CMCI method using 10 irregular cutouts from our institute's clinical database (Fig. 1). These cases were for breast patients, especially breast boost irradiation cases. Generally, the breast boost was treated with a 1–1.5 cm margin, but we intentionally reduced the margin to simulate extreme irregularity in two cases [Figure 1. (9) and (10)].

### 2.4 Using limited cutout measurements to generate the kernels

In an attempt to reduce the cutout measurements, we used the limited circular fields to generate the kernels at extended SSDs using an inverse square law. Since the effective SSD does not change significantly for larger cutout sizes, output factors with a radius greater than or equal to 4 cm were derived from an inverse square law. The effective SSD is defined as the distance from the virtual source position to the point of nominal SSD, and it can be used for small SSD differences from the nominal SSD. The output factor from an inverse square law using the effective SSD is defined as $\frac{OF_{0}}{OF_{\mathrm{ext}}}={\left(\frac{\mathrm{SS}D_{\mathrm{eff}}+d_{\max}+\mathrm{SS}D_{\mathrm{ext}}-\mathrm{SS}D_{0}}{\mathrm{SS}D_{\mathrm{eff}}+d_{\max}}\right)}^{2}$ where $OF_{0}$ and $OF_{\mathrm{ext}}$ are the output factors of circular fields at nominal SSD, SSD_0_, and extended SSD, $\mathrm{SS}D_{\mathrm{ext}}$; $\mathrm{SS}D_{\mathrm{eff}}$ is effective SSD; and $d_{\max}$ is the maximum depth.

To compare two output factors from convolution kernels with a full and limited set of circular fields with an inverse square law, we calculated a percentage differences as $\%\text{difference}=\frac{|{\text{COF}}_{f}-{\text{COF}}_{l}|}{0.5({\text{COF}}_{f}\mp {\text{COF}}_{l})}\cdot 100$ where $\mathrm{CO}F_{f}$ and COF_*l*_ are the cutout output factors from kernels with a full set and limited set of circular fields.

## 3 RESULTS

**Figure 2:**
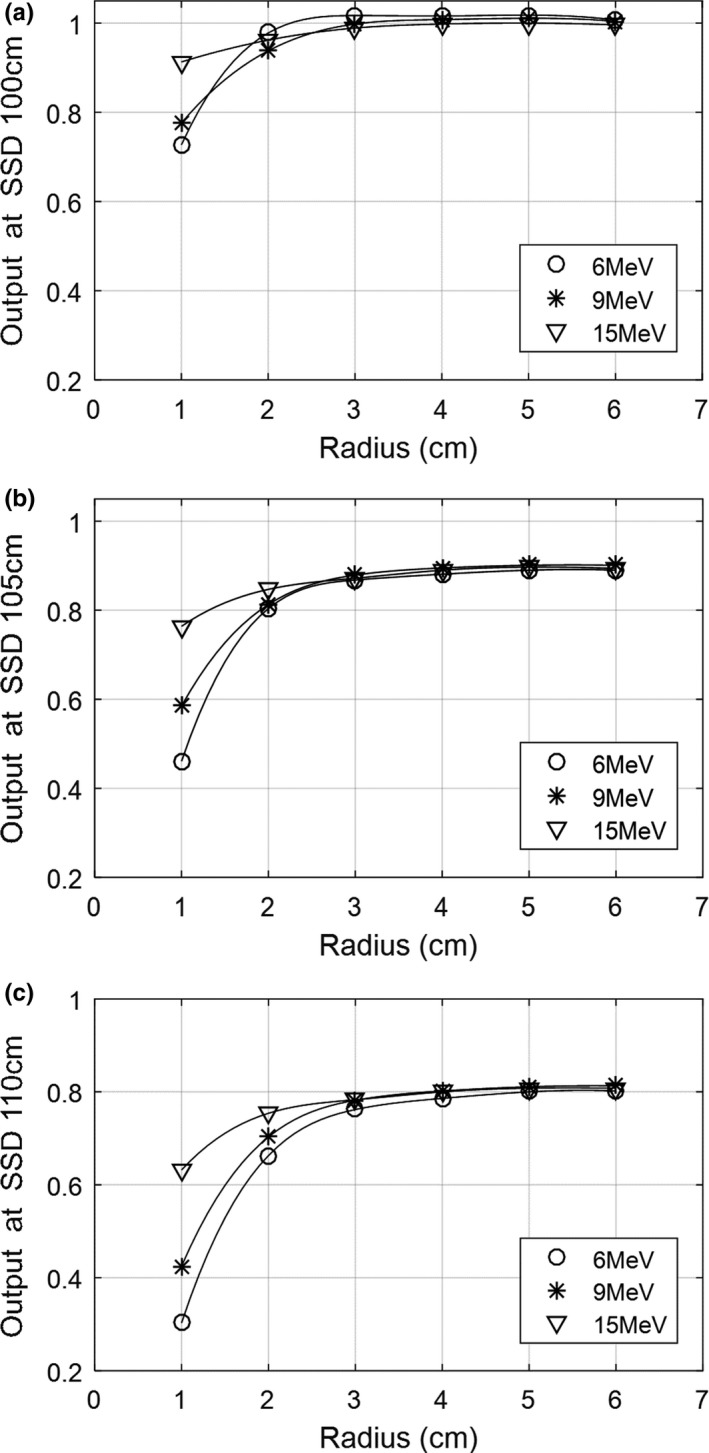
The cutout factors of circular fields at (a) nominal SSD of 100 cm and (b) extended SSD of 105 cm and (c) 110 cm.

**Figure 3:**
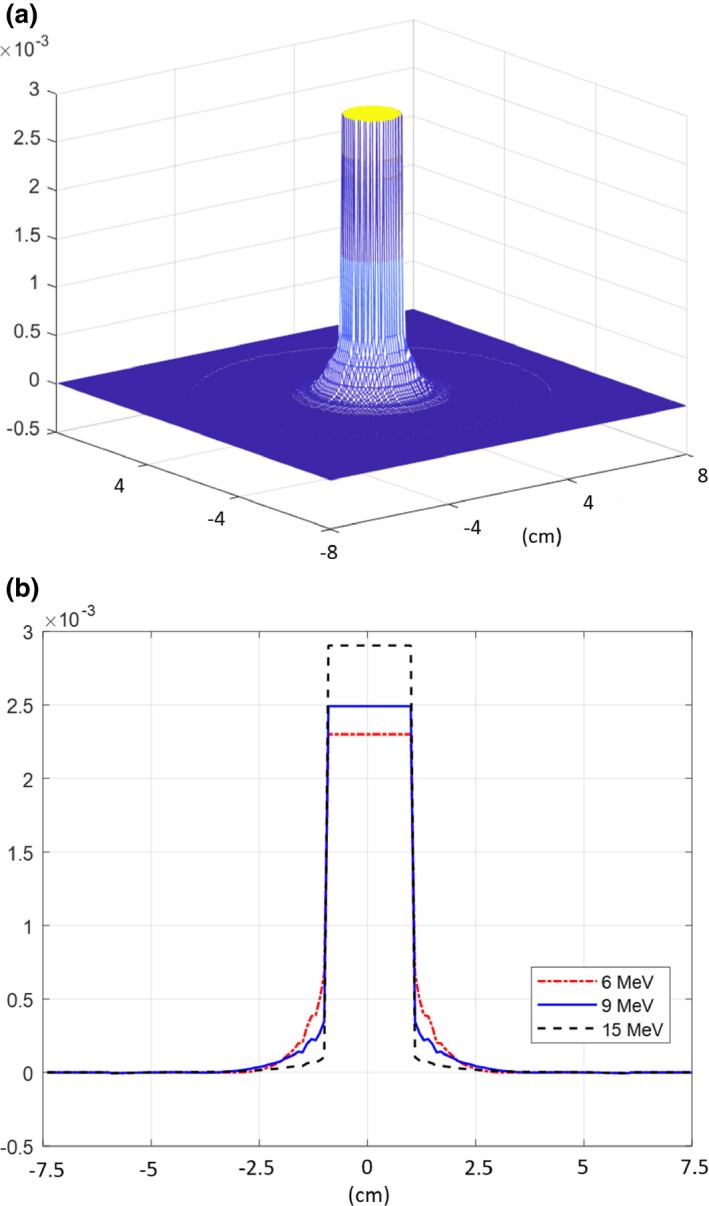
The (a) overlapped view of the convolution kernels and (b) profiles at nominal SSD of 100 cm.

The output factors of circular fields for 6E, 9E, and 15E at nominal and extended SSDs were stabilized around 4 cm and converged to 1, 0.9, and 0.8 for 6E, 9E, and 15E, respectively (Fig. 2). The overlapped view of convolution kernels and profiles at the nominal SSD of 100 cm shows that the maximum value of 0.0029 in an isotropic convolution kernel can be found at the high energy of 15E, and spreading near the edge is dominant at the low energy of 6E (Fig. 3).

### 3.1 Nominal SSD of 100 cm

At nominal SSD of 100 cm, the mean absolute errors (MAE) between the CMCI method and measurements were 0.47% (−0.66% to 1.19%), 0.67% (−1.12% to 1.48%), and 1.21 (−2.49% to 0.99%), while the errors between eMC and measurements were 0.92% (−1.39% to 1.81%), 1.17% (−1.39% to 2.3%), and 2.02% (−4.2% to 0.3%) for 6E, 9E, and 15E, respectively (Table 1). While both results showed good agreement with the chamber measurements at all energies, we found slightly larger errors at 15E.

**Table 1:** The output factor comparison result with ion chamber measurement and eMC and MAE at nominal SSD of 100 cm

Case	6 MeV					9 MeV					15 MeV				
	Meas.	CMCI	Err. (%)	eMC	Err. (%)	Meas.	CMCI	Err. (%)	eMC	Err. (%)	Meas.	CMCI	Err. (%)	eMC	Err. (%)
1	1.021	1.014	−0.66	1.008	−1.25	1.006	0.995	−1.12	0.992	−1.39	0.999	0.985	−1.37	0.979	−2.01
2	0.931	0.935	0.41	0.946	1.63	0.916	0.918	0.23	0.920	0.44	0.976	0.959	−1.70	0.948	−2.88
3	1.007	1.001	−0.57	1.009	0.23	0.980	0.976	−0.46	0.974	−0.61	1.003	0.978	−2.49	0.961	−4.20
4	1.013	1.010	−0.34	0.999	−1.39	0.988	0.987	−0.13	0.978	−1.01	0.989	0.982	−0.71	0.976	−1.32
5	1.016	1.015	−0.12	1.014	−0.15	1.013	1.004	−0.88	1.002	−1.09	1.001	0.993	−0.83	0.983	−1.80
6	0.967	0.969	0.25	0.965	−0.23	0.932	0.935	0.27	0.922	−1.07	0.965	0.961	−0.41	0.960	−0.53
7	0.971	0.983	1.19	0.989	1.81	0.945	0.959	1.48	0.954	0.95	0.964	0.974	0.99	0.967	0.30
8	0.993	0.988	−0.47	0.989	−0.44	0.969	0.964	−0.57	0.959	−1.03	0.984	0.975	−0.97	0.970	−1.43
9	0.874	0.873	−0.15	0.880	0.68	0.872	0.874	0.25	0.888	1.83	0.961	0.944	−1.74	0.932	−3.04
10	0.873	0.878	0.54	0.885	1.39	0.870	0.882	1.32	0.890	2.30	0.956	0.948	−0.85	0.931	−2.64
MAE			0.47		0.92			0.67		1.17			1.21		2.02

**Figure 4:**
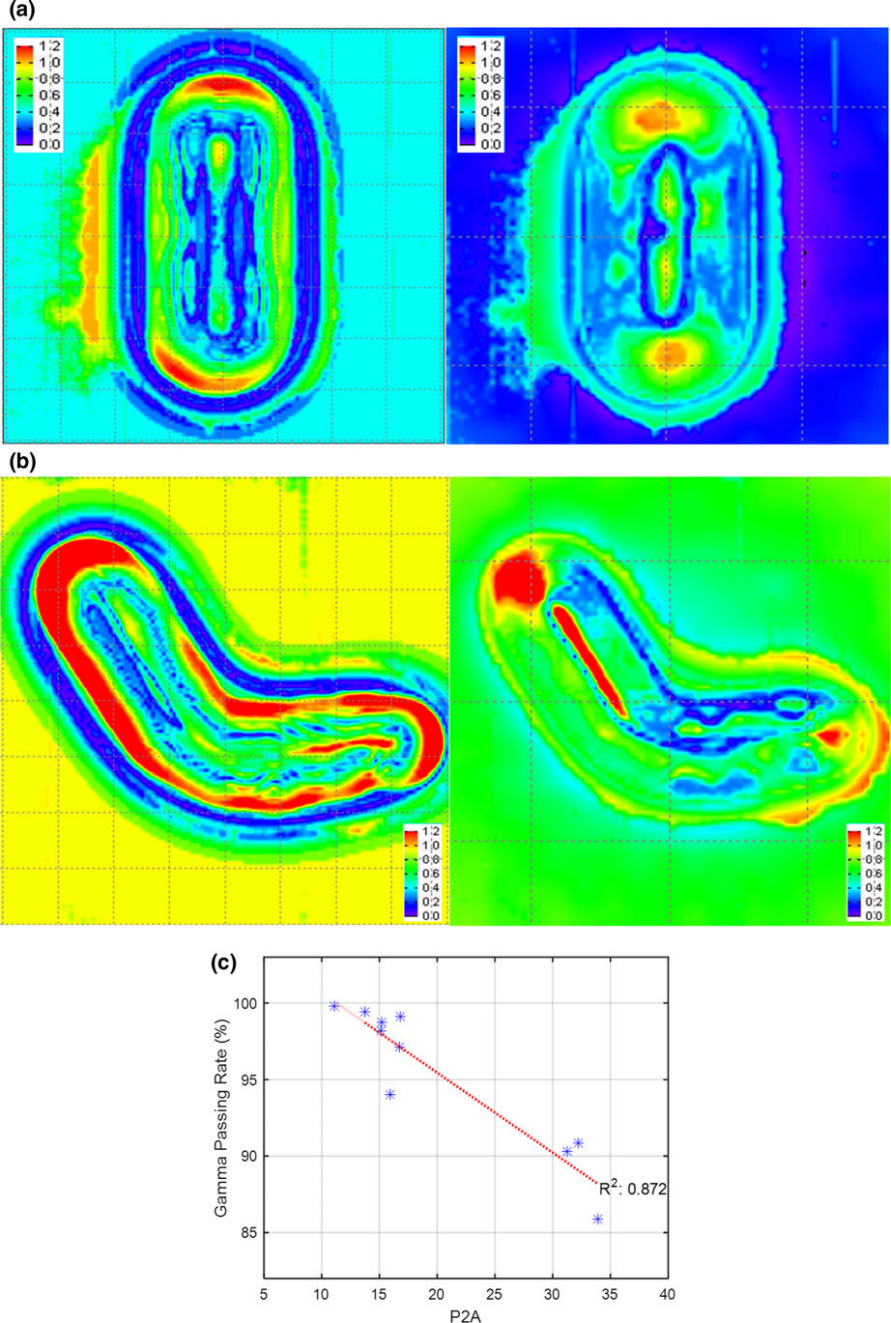
The relative output distributions of CMCI method with EDR2 film results. The relative distributions of (a) the sample case number 7 and (b) 9 were shown. The figures show the output distributions from CMCI method (left) and eMC method (right), respectively. (c) The correlation between the gamma passing rates of CMCI method and P2A (perimeter^2^/area ratio) were shown (gamma passing criteria: 3% of dose and 3 mm of DTA).

The relative output distributions of the CMCI method with EDR2 film results are presented in Fig. 4. The comparison view of the sample patient (case number 7) presented in Fig. 4(a) shows good gamma passing rates (3%/3 mm) of 97.11% for CMCI (left) and 99.02% for the eMC method (right). The comparison view of highly irregular case (case number 9) presented in Fig. 4(b) shows gamma passing rates (3%/3 mm) of 90.32% for CMCI (left) and 95.62% for the eMC method (right). Relatively large discrepancies appear at the edge of the cutout in the CMCI results because we ignored the angular dependence of scattering, based on the assumption of shift‐invariant convolution. The accuracy of a relative output distribution from the CMCI method correlates with the shape complexity measure P2A (peripery^2^/area ratio) with *R* ^2^ value of 0.872 in Fig. 4(c), while no trend was found in the eMC results. The acceptance level for IMRT plans set as a 90% gamma passing rate (the percentage of points with gamma ≤ 1.00) at 3% dose and 3 mm distance to agreement in our institute. Hence, the P2A cutoff value of 30 in Fig. 4(c) was recommended for CMCI to obtain an accurate relative dosimetry.

### 3.2 Extended SSD of 105 cm and 110 cm

At extended SSD of 105 cm, the MAEs between the CMCI method and measurements were 1.02% (−1.71% to 2.31%), 0.95% (−2.34% to 2.56%), and 0.78 (−1.39% to 1.59%), while the errors between eMC and measurements were 2.05% (−6.72% to 2.25%), 0.96% (−2.07% to 3.10%), and 1.25% (−2.47% to 1.25%) for 6E, 9E, and 15E, respectively (Table 2). The results showed that the CMCI method generated more accurate output factors compared to eMC results at SSD of 105 cm.

**Table 2:** The cutout output factor comparison result with ion chamber measurement and eMC and MAE at nominal SSD of 105 cm

Case	6 MeV					9 MeV					15 MeV				
	Meas.	CMCI	Err. (%)	eMC	Err. (%)	Meas.	CMCI	Err. (%)	eMC	Err. (%)	Meas.	CMCI	Err. (%)	eMC	Err. (%)
1	0.886	0.873	−1.51	0.882	−0.40	0.889	0.880	−1.07	0.890	0.13	0.883	0.877	−0.68	0.871	−1.40
2	0.771	0.776	0.60	0.759	−1.60	0.784	0.785	0.10	0.792	1.08	0.845	0.840	−0.59	0.842	−0.41
3	0.860	0.855	−0.57	0.867	0.82	0.857	0.857	0.01	0.848	−1.00	0.872	0.868	−0.50	0.855	−2.00
4	0.874	0.864	−1.11	0.867	−0.79	0.878	0.869	−1.07	0.870	−0.94	0.877	0.872	−0.58	0.859	−2.10
5	0.887	0.880	−0.83	0.892	0.52	0.899	0.891	−0.90	0.900	0.15	0.891	0.885	−0.65	0.883	−0.94
6	0.811	0.810	−0.16	0.797	−1.79	0.810	0.807	−0.37	0.809	−0.15	0.850	0.845	−0.61	0.848	−0.29
7	0.814	0.833	2.31	0.832	2.25	0.815	0.836	2.56	0.840	3.10	0.847	0.861	1.59	0.858	1.25
8	0.848	0.841	−0.80	0.840	−0.89	0.845	0.844	−0.13	0.843	−0.20	0.864	0.863	−0.14	0.863	−0.16
9	0.694	0.698	0.56	0.662	−4.68	0.730	0.723	−0.97	0.736	0.75	0.824	0.816	−1.02	0.811	−1.52
10	0.718	0.706	−1.71	0.670	−6.72	0.749	0.732	−2.34	0.733	−2.07	0.832	0.820	−1.39	0.811	−2.47
MAE			1.02		2.05			0.95		0.96			0.78		1.25

At extended SSD of 110 cm, the MAEs between the CMCI method and measurements were 1.73% (−5.73% to 1.96%), 1.00% (−2.61% to 2.78%), and 0.68 (−1.44% to 1.62%), while the errors between eMC and measurements were 3.30% (1.86% to −9.26%), 0.94% (−3.19% to 2.44%), and 1.09% (−2.48% to 1.63%) for 6E, 9E, and 15E, respectively (Table 3). The larger errors were found in the highly irregular field in both methods. However, eMC generated more significant errors than the CMCI method, showing more than 5% (up to −9.28%) error in three test cases.

**Table 3:** The cutout output factor comparison result with ion chamber measurement and eMC and MAE at nominal SSD of 110 cm

Case	6 MeV					9 MeV					15 MeV				
	Meas.	CMCI	Err. (%)	eMC	Err. (%)	Meas.	CMCI	Err. (%)	eMC	Err. (%)	Meas.	CMCI	Err. (%)	eMC	Err. (%)
1	0.776	0.764	−1.52	0.773	−0.39	0.790	0.784	−0.82	0.787	−0.33	0.792	0.788	−0.47	0.783	−1.09
2	0.622	0.618	−0.61	0.594	−4.49	0.660	0.669	1.36	0.663	0.50	0.744	0.741	−0.44	0.741	−0.37
3	0.730	0.730	0.03	0.738	1.13	0.750	0.757	0.87	0.747	−0.44	0.775	0.777	0.30	0.765	−1.25
4	0.757	0.747	−1.32	0.753	−0.59	0.775	0.770	−0.65	0.770	−0.63	0.784	0.782	−0.23	0.776	−0.98
5	0.783	0.781	−0.20	0.798	1.86	0.801	0.798	−0.41	0.808	0.84	0.801	0.797	−0.56	0.795	−0.70
6	0.662	0.653	−1.33	0.627	−5.32	0.695	0.696	0.07	0.694	−0.17	0.753	0.750	−0.36	0.754	0.17
7	0.684	0.697	1.96	0.692	1.20	0.711	0.731	2.78	0.728	2.44	0.755	0.767	1.62	0.767	1.63
8	0.720	0.710	−1.35	0.703	−2.29	0.739	0.741	0.26	0.743	0.49	0.769	0.771	0.31	0.773	0.56
9	0.539	0.522	−3.21	0.504	−6.48	0.595	0.594	−0.20	0.593	−0.32	0.714	0.706	−1.06	0.702	−1.67
10	0.567	0.535	−5.73	0.514	−9.29	0.620	0.604	−2.61	0.600	−3.19	0.722	0.712	−1.44	0.704	−2.48
MAE			1.73		3.30			1.00		0.94			0.68		1.09

We found larger errors at 6E in eMC results because of an innate problem at low‐energy beam (≤6E), which we will discuss later. Because of its measurement basis, the CMCI method generated accurate results even at a low‐energy beam, except in two highly irregular fields.

### 3.3 Results from kernels with limited cutout measurements

**Figure 5:**
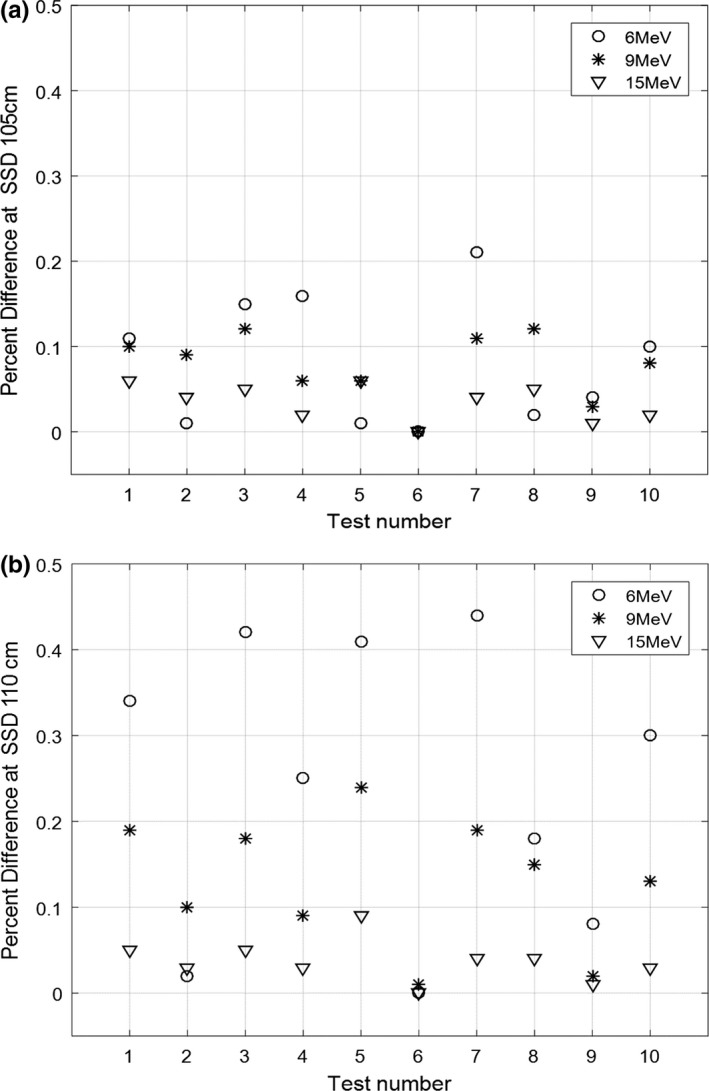
The percentage differences between results from the kernels with a full and limited set of circular fields at 6E, 9E, and 15E at extended SSD of (a) 105 cm and (b) 110 cm.

Comparing results from the kernels with a full and limited set of circular fields, the mean absolute percentage differences at SSD of 105 cm were 0.082%, 0.076%, and 0.033%, for 6E, 9E, and 15E, respectively [Fig. 5(a)]. At SSD of 110 cm, these values were 0.243%, 0.132%, and 0.036% for 6E, 9E, and 15E, respectively [Fig. 5(b)]. The percentage differences were relatively larger (up to −0.45%) at 6E at extended SSDs. Since effective SSD does change with energy, especially for low energies, incorrect circular cutout factors derived by an inverse square law may cause relatively larger errors. However, overall results from the kernel of limited circular fields were compatible with those from the kernel of full circular fields set, showing that the absolute mean and standard deviation of all percentage differences are 0.06 ± 0.05% for SSD of 105 cm and 0.14 ± 0.13% for SSD of 110 cm.

## 4 DISCUSSION

We presented a new CMCI method for predicting an electron cutout factor, especially for irregularly shaped cutouts. In contrast to the original Clarkson integration method, our method used annular sectors to estimate the output factor, and its outcome is a 2D distribution, which offers the relative output distribution as well as the output factor at the specific point. The CMCI method produced slightly larger errors in results at 6E at an extended SSD of 110 cm than at other electron beam energies and SSDs. For highly irregular fields at lower energy electron beams, the background convolution assumptions of system linearity and shift invariance may not be fulfilled because of uncertainties related to larger scatter angles of low‐energy incident beams and dependence on an interaction angle between the central beams the vertical edge of a cutout. Hence, CMCI method may not calculate accurate output when the chamber measurement point is shifted from a center area due to the differences in an interaction angle between central beams and the vertical edge of a cutout because 2D convolution output kernel was generated using measurement values of circular cutouts at the center. However, these errors are insignificant in clinical cutout shapes and ranges. Except for test cases 9 and 10, in which we intentionally reduced the margin to simulate extreme irregularity in our study, the differences between CMCI and measurements were within ± 2.5%. Our CMCI method proved effective in terms of accuracy, calculation time, and area covered for a single calculation.

Known limitations for eMC calculation of electron beams with energies ≤6E include^16^ differences in up to 5% between measured and calculated outputs for 6E electron beams and differences in up to 14% for circular inserts with a diameter of 3 cm at an extended SSD of 115 cm.^17^ Approximations of the electron path in the direction distribution and dose deposition determine whether large sphere sizes for the electron transport are used in eMC.^18^ However, several studies demonstrate that eMC can predict dose distributions for high‐energy electron beams with high accuracy.^16^, ^19^, ^20^ This explains why we observed large errors at 6E electrons at extended SSDs. However, eMC's overall calculation accuracy showed good agreement with the measured values of the other electron beam energies without showing a specific trend related to energy and cutout shape.

One of our convolution method's strengths is its ability to detect a maximum cutout output value as it calculates the 2D output distribution. The convolution maximum point is useful when the ion chamber measurement is required. In the clinic, using irregular cutouts with an extended SSD setting requires taking chamber measurements. Ideally, a measurement should be performed at the expected maximum dose point, but such measurements are prone to point variability, particularly for narrow and irregular cutout fields. Our convolution method offers a stable position for determining an ion chamber measurement point by offering a convolution maximum point.

In this study, we tested our method for the 15 × 15 cone size as a proof of concept. One drawback of the CMCI method is that it requires multiple circular cutout factor measurements for different energies, cone sizes, and SSD settings. In this study, our model was built based on four cutout measurements (ranging from 3 to 6 cm) at three beam energies and SSDs. To solve this issue, we evaluated the convolution kernels from a limited set of circular fields with an inverse square law using an effective SSD and verified them by comparing percentage differences between results from convolution kernels with a full and limited set of circular fields. The results were promising, and we are continuing to investigate the minimum measurements required to obtain reliable results and comparable kernels at large sizes (15 × 15, 20 × 20, 25 × 25) and high electron beam energies.

## 5 CONCLUSION

We have developed an efficient and accurate model for predicting electron cutout outputs for arbitrary‐shaped cutouts. Our CMCI method efficiently and accurately calculates entire 2D distributions of cutout factors. Our method can generate comparable results to the eMC method at clinically used cutout settings and can be used for the second MU verification calculation.

## CONFLICT OF INTEREST

No conflict of interest.
